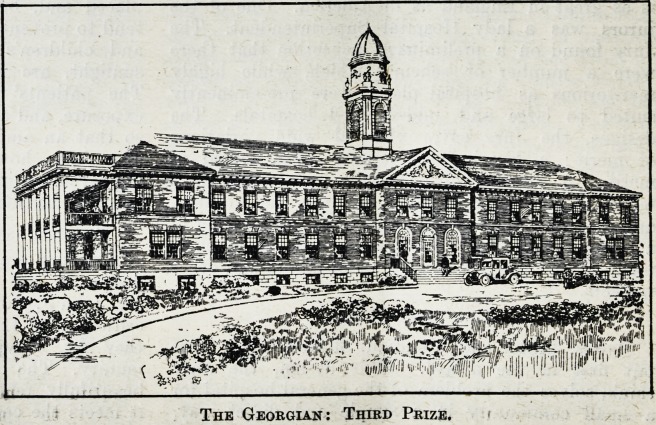# The American Small Hospital

**Published:** 1924-01

**Authors:** 


### THE AMERICAN SMALL HOSPITAL.

### A COMPETITION IN DESIGN AND PLANNING.

THE *Modern Hospital* of Chicago recently instituted an architectural competition for a small hospital of from thirty to forty beds. The object was "to stimulate a greater interest in the construction of an economically arranged and architecturally artistic small hospital, and to provide Boards of Trustees of small institutions with a group of suggestive plans that will make for more efficient care and treatment of the sick." The result has been published in a very fully illustrated volume ("Architectural Designs for a Small Hospital with Suggestions on Organisation and Equipment": Chicago, *The Modern Hospital*, 1 dollar), which contains the plans and elevations not only of the prize designs and those which received honourable mention, but a selection of fifteen other competitive sets. The Chairman of the Jury of Award was Dr. S. S. Goldwater, Director of the Mount Sinai Hospital, New York, whose distinction as a hospital specialist is as great in England as in America. One of the jurors was a lady Hospital Superintendent. The Jury found on a preliminary inspection that there were a number of schemes which, while highly meritorious as hospital plans, were pre-eminently suited to large and middle-sized hospitals. The designs, the Jury say, exhibited wide variations of merit; a few in fact seemed quite devoid of merit, A number of otherwise interesting plans failed to command approval because the working, and especially the nursing units, were put together in a manner that would have compelled the employment of an unusually large force of nurses and other workers.

First Prize: "Somewhat Meagre in its Elements."

### The First Prize.

Messrs. Butler and Rodman, of New York, were put first in the award. Their design, the Jury think, solves the problem of the general hospital for a small community in a simple and direct way, making for economy of construction. The design, while somewhat meagre in its elements, is altogether appropriate and satisfactory, and has little of the institutional look. The general shape of the building and the pleasant skyline should give, with well chosen materials, a very satisfactory effect. The construction is very simple and economical, and indicates the use of materials and workmanship easily procurable in almost any locality. Each department can be approached without disturbing the activities of any other department. The kitchen and other important service portions are, on the whole, well arranged and occupy well lighted rooms. The lifts and staircases, the ambulance and supply approaches and various work rooms are so placed that their use will not disturb the patients. This can also be said of the emergency, receiving and general operating rooms. The nursing and housekeeping arrangements of the upper floor are well placed and, being simply arranged on each floor, tend to prevent confusion in service. The maternity and children's departments, while having ample sunlight, are well separated from other patients. The patients' rooms are all placed with sunny exposure, and are of various sizes and appointments, so that an ample variety of service may be given. The Jury, however, make several suggestions for improvement in the plans from the point of view of practical hospital administration.

### The Second Place: Italian Renaissance.

The Jury are enthusiastic about the architectural design of the second premiated set, the work of Mr. John J. Roth, of Los Angeles, though somewhat critical upon the details of the plan. It represents, they say, a type very suitable for the small community. The exterior is simple, pleasing and very beautifully rendered. In economy of construction it meets the conditions well, although simplification could be made without injury to the plan. The location of departments is made with intelligence, and makes for efficiency of operation and economy in nursing service. The patients in general are well provided with sunlight. Certain sections of the basement, however, are poorly lighted and ventilated. In the basement, moreover, all the corridors are blocked from light and air. The design of the exterior is unique and refreshing, in that attention is given to form and colour to an unusual degree. Undoubtedly it would be a distinctive building if erected in a proper environment.

Second Prize: Italian Renaissance.

Two Storeyed Georgian: Third Prize.

The third prize design, by Mr. Ernest C. Hoedtke, of Cambridge, Mass., is in the Georgian manner. The Jury describe it as a very excellent plan, although somewhat more ambitious and expensive than the competition warrants. The general construction of the building is simple, but it is, perhaps, a bit too severe and institutional in its handling, and the central tower seems out of proportion to the rest of the building and an unnecessarily expensive feature. The relation of the departments one to another is good, and efficiency of operation was given careful study by the competitor. Patients are well taken care of in sunny rooms, with ample porch space. The nursing and medical functions are well handled. It is apparent from the refinements of detail shown on the drawing that the designer would be capable of making a thoroughly interesting design of this building if he were to give it more study. The plan lends itself very well to enlargement. The competitor's presentation of the problem is one of the best, and would have been entitled to a higher rating had the design been given more study from an architectural point of view, and had it more thoroughly embodied the idea of a small compact institution. It will thus be seen that the Jury were by no means unconscious of the practical shortcomings of even the selected designs.

The Georgian: Third Prize.

## Figures and Tables

**Figure f1:**
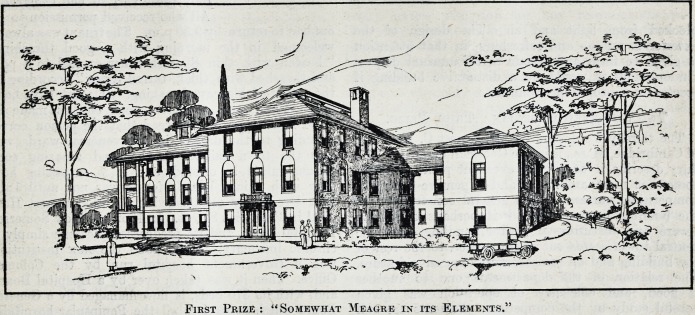


**Figure f2:**
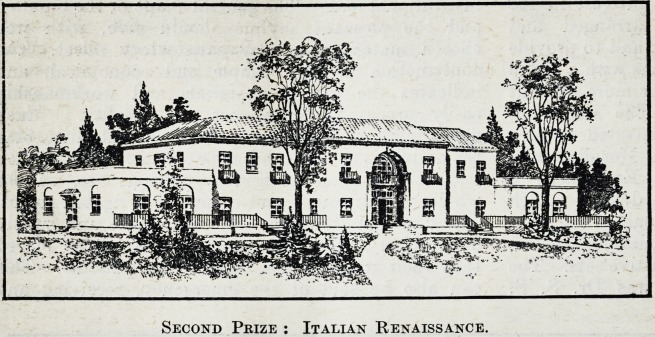


**Figure f3:**